# Adverse outcomes after noncardiac surgery in patients with aortic stenosis

**DOI:** 10.1038/s41598-021-98507-6

**Published:** 2021-09-30

**Authors:** Ying-Hsuan Tai, Chuen-Chau Chang, Chun-Chieh Yeh, Yih-Giun Cherng, Ta-Liang Chen, Chien-Chang Liao

**Affiliations:** 1 Department of Anesthesiology, Shuang Ho Hospital, Taipei Medical University, New Taipei City, Taiwan; 2 Department of Anesthesiology, School of Medicine, College of Medicine, Taipei Medical University, Taipei, Taiwan; 3 Department of Anesthesiology, Taipei Medical University Hospital, 252 Wuxing St., Taipei, 110 Taiwan; 4 Anesthesiology and Health Policy Research Center, Taipei Medical University Hospital, Taipei, Taiwan; 5 Department of Surgery, China Medical University Hospital, Taichung, Taiwan; 6 Department of Surgery, University of Illinois, Chicago, IL USA; 7 Department of Anesthesiology, Wan Fang Hospital, Taipei Medical University, Taipei, Taiwan; 8 Research Center of Big Data and Meta-Analysis, Wan Fang Hospital, Taipei Medical University, Taipei, Taiwan; 9 School of Chinese Medicine, College of Chinese Medicine, China Medical University, Taichung, Taiwan

**Keywords:** Cardiology, Diseases, Health care

## Abstract

Whether aortic stenosis (AS) increases perioperative risk in noncardiac surgery remains controversial. Limited information is available regarding adequate anesthetic techniques for patients with AS. Using the reimbursement claims data of Taiwan’s National Health Insurance, we performed propensity score matching analyses to evaluate the risk of adverse outcomes in patients with or without AS undergoing noncardiac surgery between 2008 and 2013. We also compared the perioperative risk of AS patients undergoing general anesthesia or neuraxial anesthesia. Multivariable logistic regressions were applied to calculate the adjusted odds ratios (aORs) with 95% confidence intervals (CIs) for postoperative mortality and major complications. The matching procedure generated 9741 matched pairs for analyses. AS was significantly associated with 30-day in-hospital mortality (aOR 1.31, 95% CI 1.03–1.67), acute renal failure (aOR 1.42, 95% CI 1.12–1.79), pneumonia (aOR 1.16, 95% CI 1.02–1.33), stroke (aOR 1.14, 95% CI 1.01–1.29), and intensive care unit stay (aOR 1.38, 95% CI 1.27–1.49). Compared with neuraxial anesthesia, general anesthesia was associated with increased risks of acute myocardial infarction (aOR 3.06, 95% CI 1.22–7.67), pneumonia (aOR 1.80, 95% CI 1.32–2.46), acute renal failure (aOR 1.82, 95% CI 1.11–2.98), and intensive care (aOR 4.05, 95% CI 3.23–5.09). The findings were generally consistent across subgroups. AS was an independent risk factor for adverse events after noncardiac surgery. In addition, general anesthesia was associated with greater postoperative complications in AS patients compared to neuraxial anesthesia. This real-world evidence suggests that neuraxial anesthesia should not be contraindicated in patients with AS.

## Introduction

Aortic stenosis (AS), the most common valvular heart disease in developed countries, affects approximately 12 million individuals worldwide and causes a substantial and growing burden of disease in the aging population^1–3^. Patients with AS are susceptible to perioperative cardiac complications due to an unfavorable hemodynamic state from anesthetics and surgical stress. Current guidelines recommend that surgical or percutaneous interventions be performed for patients with severe or symptomatic AS prior to elective noncardiac surgery^4, 5^, which is primarily based on the studies indicating AS a major risk factor for mortality and adverse cardiac events after noncardiac surgery^6–17^; however, the association between AS and perioperative risk has not been well clarified because of conflicting results and study limitations, such as small numbers of AS patients (< 1000)^6–10,12–15,17^, inadequate control for confounding factors^7–9^, an absence of non-AS controls^7,9,17^, and restriction to specific populations or hospitals^6–10,12–15,17^. In addition, some previous studies were based on data that are now more than two decades old, which can hardly reflect the recent advances in surgical and anesthetic techniques for AS patients^6–11^.

Neuraxial anesthesia has long been regarded as a contraindication for patients with AS due to its sympatholytic effect, potentially causing precipitous falls in vascular resistance and thereafter reduced cardiac output and coronary perfusion^18^; however, the concern related to neuraxial anesthesia use in AS patients is borne of a theoretical risk rather than clinical evidence^19,20^. Only a few case reports have described neuraxial anesthesia use in AS patients^21–23^, and no large-scale study comparing the perioperative risk of AS patients undergoing general or neuraxial anesthesia has been reported. In general, anesthesia, volatile and intravenous anesthetics tend to reduce sinus node automaticity and may cause the loss of atrioventricular synchrony, arrhythmias, myocardial depression, and heart failure^24^. Further, general anesthesia may increase respiratory complications and 30-day mortality in patients with high cardiac risk compared to regional anesthesia^25,26^.

Accordingly, we conducted a population-based cohort study to examine the risk of noncardiac surgery in patients with AS. In addition, considering the lack of evidence pertaining to safer anesthetic techniques for AS patients, we also compared the risk of adverse outcomes between general and neuraxial anesthesia use in AS patients. Based on previous evidence^6–17,21–26^, we hypothesized that AS is independently associated with higher risks of adverse outcomes and general anesthesia carries a higher perioperative risk compared to neuraxial anesthesia for AS patients undergoing noncardiac surgery.

## Methods

### Source of data

Since the Taiwan National Health Insurance program was implemented in March 1995, more than 99% of the 23 million residents have participated in this health program. This study used the reimbursement claims data from Taiwan’s National Health Insurance that included information of beneficiaries’ medical services, such as inpatient and outpatient demographic characteristics, physicians’ primary and secondary diagnoses, treatment procedures, prescriptions, and medical expenditures. Research articles based on this database and the related validation have been accepted in prominent scientific journals worldwide^27–29^.

### Ethical approval

This study was conducted in accordance with the Helsinki Declaration. To protect personal privacy, the electronic database was decoded with patient identifications scrambled for further research. According to the regulations from Ministry of Health and Welfare, informed consent is not required because of the use of decoded and scrambled patient identifications. This study was reviewed and approved by the Joint Institutional Review Board of Taipei Medical University (TMU-JIRB-202002026; TMU-JIRB-201905042; TMU-JIRB-201902053). The Joint Institutional Review Boards of Taipei Medical University waived the need for informed consent of this study. All methods were carried out in accordance with relevant guidelines and regulations.

### Study design

Among 3.5 million surgical patients aged more than 20 years who underwent noncardiac inpatient surgeries in Taiwan in 2008–2013, we examined medical claims and identified 13,251 people who had history of AS within 24 months prior to the index surgery. Each patient with AS who underwent noncardiac surgery was randomly matched to a surgical patient who had no history of AS, using a frequency matched pair procedure (case–control ratio, 1:1) to adjust for age, sex, income status, types of anesthesia, types of surgery, medical conditions, hospitalization experiences and emergency visits. A total of 9,741 matched pairs were included as eligible subjects for comparison. The purpose of this study was to investigate whether AS is a risk factor for postoperative adverse outcomes.

From the 9,741 surgical patients with AS, we excluded 4,710 patients who underwent surgical procedures that could only be performed with general anesthesia, such as neurological, pulmonary, breast, head and neck, etc. We compared the postoperative complications and mortality between the 5031 patients with AS who underwent surgeries that were performed with neuraxial or general anesthesia to investigate whether general anesthesia had increased adverse surgical outcomes.

### Definition and criteria

Based on our previous study^27,28^, a low-income status was defined by qualifying for waived medical copayment as verified by the Ministry of Health and Welfare, Taiwan. The International Classification of Diseases, Ninth Revision, Clinical Modification (ICD-9-CM) codes and physicians’ diagnoses were used to identify the history of disease (within preoperative 24 months) and postoperative complications (that occurred during the index admission) for surgical patients. These medical conditions were determined from medical claims for the 24-month preoperative period, including hypertension (ICD-9-CM 401–405), ischemic heart disease (ICD-9-CM 410–414), mental disorders (ICD-9-CM 290–319), diabetes (ICD-9-CM 250), chronic obstructive pulmonary diseases (ICD-9-CM 490, 491, 496), heart failure (ICD-9-CM 428), hyperlipidemia (ICD-9-CM 272.0, 272.1, 272.2, 272.4), renal dialysis (administration code D8, D9), and liver cirrhosis (ICD-9-CM 571.2, 571.5, 571.6). Postoperative complications were determined from medical claims that occurred during the index admission, including postoperative bleeding, pneumonia, septicemia, urinary tract infection, deep wound infection, stroke, acute myocardial infarction, acute renal failure and pulmonary embolism. A requirement for intensive care, the length of hospital stay and medical expenditures during the index surgical admission were also compared between patients with and without AS. In this study, we defined intensive care unit stay as patients during the surgical admission who received intensive care after surgery.

### Statistical analysis

For the determination of the relationships between AS and the postoperative outcomes, we used a nonparsimonious multivariable logistic regression model to estimate a propensity score for each of the surgical patients with AS or without AS. Clinical significance guided the initial choice of the covariates in this model to include age groups (20–29, 30–39, 40–49, 50–59, 60–69, 70–79, and ≥ 80 years), sex, income status (low income or not), types of surgery, types of anesthesia, hypertension, ischemic heart disease, mental disorders, diabetes, chronic obstructive pulmonary diseases, heart failure, hyperlipidemia, renal dialysis, liver cirrhosis, emergency visits (0, 1, and ≥ 2 visits), and hospitalizations (0, 1, and ≥ 2 visits). We matched patients with AS to non-AS controls by using a greedy matching algorithm (without replacement) with a caliper width of 0.2 standard deviations of the log odds of the estimated propensity score.

Categorical variables were summarized using frequencies (percentages) and compared between people with and without AS using chi-square tests. Continuous variables were summarized using the mean ± standard deviation and compared using t-tests. Adjusted odds ratios (ORs) and 95% confidence intervals (CIs) of postoperative complications and mortality associated with AS were calculated by multivariable logistic regressions (adjusted for age groups, sex, income status, types of surgery, types of anesthesia, hypertension, ischemic heart disease, mental disorders, diabetes, chronic obstructive pulmonary diseases, heart failure, hyperlipidemia, renal dialysis, liver cirrhosis, emergency visits, and hospitalizations). Subgroup analyses by age group (20–49, 50–59, 60–69, 70–79, and ≥ 80 years), sex (female, male), number of medical conditions (0, 1, 2, ≥ 3 medical conditions), emergency care (0, 1, ≥ 2 visits) and inpatient care (0, 1, ≥ 2 visits) were also performed to examine surgical outcomes analyzed by multivariate logistic regressions among patients with AS within these subgroups.

For comparing the risk of postoperative adverse outcomes between AS patients with general anaesthesia and neuraxial anaesthesia, we calculated adjusted ORs and 95% CIs by using multivariable logistic regressions (adjusted for age groups, sex, income status, types of surgery, types of anesthesia, hypertension, ischemic heart disease, mental disorders, diabetes, chronic obstructive pulmonary diseases, heart failure, hyperlipidemia, renal dialysis, liver cirrhosis, emergency visits, and hospitalizations). In the stratified analysis by medical conditions (0, 1, 2, and ≥ 3 medical conditions), we calculated the adjusted ORs and 95% CIs of postoperative adverse events associated with general anesthesia in the multivariable logistic regressions.

## Results

Under the frequency matching procedure, Table 1 shows the balanced distribution of age, sex, low income, hypertension, ischemic heart disease, mental disorders, diabetes, chronic obstructive pulmonary diseases, heart failure, hyperlipidemia, renal dialysis, liver cirrhosis, emergency visits, and hospitalizations between surgical patients with and without AS.

**Table 1:** Characteristics of patients with aortic stenosis received non-cardiac surgeries (after matching).

	No aortic stenosis (N = 9,741)		Aortic stenosis (N = 9,741)		*P*
**Sex**	n	(%)	n	(%)	1.0000
Female	5182	(53.2)	5182	(53.2)	
Male	4559	(46.8)	4559	(46.8)	
**Age, years**					1.0000
20–29	77	(0.8)	77	(0.8)	
30–39	163	(1.7)	163	(1.7)	
40–49	325	(3.3)	325	(3.3)	
50–59	735	(7.6)	735	(7.6)	
60–69	1646	(16.9)	1646	(16.9)	
70–79	3471	(35.6)	3471	(35.6)	
≥ 80	3324	(34.1)	3324	(34.1)	
**Low income**					1.0000
No	9680	(99.4)	9680	(99.4)	
Yes	61	(0.6)	61	(0.6)	
**Types of anesthesia**					1.0000
General	6821	(70.0)	6821	(70.0)	
Epidural or spinal	2920	(30.0)	2920	(30.0)	
**Types of surgery**					1.0000
Skin	83	(0.9)	83	(0.9)	
Breast	58	(0.6)	58	(0.6)	
Musculoskeletal	3665	(37.6)	3665	(37.6)	
Respiratory	303	(3.1)	303	(3.1)	
Digestive	2525	(25.9)	2525	(25.9)	
Kidney, ureter, bladder	737	(7.6)	737	(7.6)	
Delivery, CS, abortion	89	(0.9)	89	(0.9)	
Neurosurgery	1234	(12.7)	1234	(12.7)	
Eye	59	(0.6)	59	(0.6)	
Others	988	(10.1)	988	(10.1)	
**Number of hospitalizations**					1.0000
0	4554	(46.8)	4554	(46.8)	
1	2552	(26.2)	2552	(26.2)	
≥ 2	2635	(27.1)	2635	(27.1)	
**Number of emergency visits**					1.0000
0	4412	(45.3)	4412	(45.3)	
1	2166	(22.2)	2166	(22.2)	
≥ 2	3163	(32.5)	3163	(32.5)	
**Medical conditions**					
Hypertension	4173	(42.8)	4173	(42.8)	1.0000
Ischemic heart disease	2066	(21.2)	2066	(21.2)	1.0000
Mental disorders	2048	(21.0)	2048	(21.0)	1.0000
Diabetes	1392	(14.3)	1392	(14.3)	1.0000
COPD	1221	(12.5)	1221	(12.5)	1.0000
Heart failure	917	(9.4)	917	(9.4)	1.0000
Hyperlipidemia	324	(3.3)	324	(3.3)	1.0000
Renal dialysis	146	(1.5)	146	(1.5)	1.0000
Liver cirrhosis	108	(1.1)	108	(1.1)	1.0000

*COPD* chronic obstructive pulmonary disease, *CS* cesarean section.

After adjustment in multivariable logistic regressions (Table 2), patients with AS had higher risks of postoperative bleeding (aOR 1.94, 95% CI 1.33–2.84), acute renal failure (aOR 1.42, 95% CI 1.12–1.79), pneumonia (aOR 1.16, 95% CI 1.02–1.33), stroke (aOR 1.14, 95% CI 1.01–1.29), and 30-day mortality (aOR 1.31, 95% CI 1.03–1.67) compared to the non-AS group. A history of AS was associated with an increased risk of intensive care after noncardiac surgery (aOR 1.38, 95% CI 1.27–1.49). The higher medical expenditures (median 2410 vs. median 2283 US dollars, *p* < 0.0001) were also noted for patients with AS compared to those without AS.

**Table 2:** Adverse outcomes after non-cardiac surgeries in patients with aortic stenosis (after matching).

	No aortic stenosis (N = 9,741)		Aortic stenosis (N = 9,741)		Risk of outcomes	
	Events	%	Event	%	aOR	(95% CI)^a^
30-day in-hospital mortality	122	1.3	158	1.6	1.31	(1.03–1.67)
**Postoperative complications**						
Pulmonary embolism	23	0.2	25	0.3	1.09	(0.62–1.92)
Acute myocardial infarction	43	0.4	42	0.4	0.98	(0.64–1.50)
Acute renal failure	128	1.3	180	1.9	1.42	(1.12–1.79)
Pneumonia	457	4.7	523	5.4	1.16	(1.02–1.33)
Septicemia	629	6.5	627	6.4	1.00	(0.89–1.12)
Stroke	560	5.8	630	6.5	1.14	(1.01–1.29)
Urinary tract infection	792	8.1	772	7.9	0.97	(0.87–1.08)
Postoperative bleeding	41	0.4	79	0.8	1.94	(1.33–2.84)
Deep wound infection	38	0.4	38	0.4	1.00	(0.64–1.57)
ICU stay	1724	17.7	2118	21.7	1.38	(1.27–1.49)
Medical expenditure, USD^†^	2283 (3107)		2410 (3114)		p < 0.0001	
Length of hospital stay, days^†^	7.0 (8.0)		7.0 (9.0)		p < 0.0001	

*CI* confidence interval, *aOR* adjusted odds ratio.

^a^Adjusted for all covariates listed in Table 1.

^†^Median (interquartile range).

Among 5,031 AS patients who underwent surgery with general or neuraxial anesthesia (Table 3), those who underwent general anesthesia had lower proportions of male (*p* < 0.0001), older people ≥ 80 years of age (*p* < 0.0001), musculoskeletal surgery (*p* < 0.0001), hypertension (*p* = 0.0002), ischemic heart disease (*p* < 0.0001), and chronic obstructive pulmonary disease (*p* = 0.0009) compared with those who underwent neuraxial anesthesia.

**Table 3:** Characteristics of patients with aortic stenosis who received surgeries required neuraxial and general anesthesia.

	NA (N = 3,011)		GA (N = 2,020)		*P*
	n	(%)	n	(%)	
**Sex**					< 0.0001
Female	1313	(43.6)	1037	(51.3)	
Male	1698	(56.4)	983	(48.7)	
**Age, years**					
20–29	26	(0.9)	10	(0.5)	< 0.0001
30–39	74	(2.5)	45	(2.2)	
40–49	58	(1.9)	89	(4.4)	
50–59	144	(4.8)	167	(8.3)	
60–69	436	(14.5)	367	(18.2)	
70–79	1013	(33.6)	715	(35.4)	
≥ 80	1260	(41.9)	627	(31.0)	
**Low income**					0.0458
No	2947	(97.9)	1959	(97.0)	
Yes	64	(2.1)	61	(3.0)	
**Types of surgery**					< 0.0001
Musculoskeletal	1414	(47.0)	870	(43.1)	
Digestive	602	(20.0)	357	(17.7)	
Kidney, ureter, bladder	498	(16.5)	474	(23.5)	
Delivery, CS, abortion	82	(2.7)	20	(1.0)	
Others	415	(13.8)	299	(14.8)	
**Number of hospitalizations**					0.0704
0	1358	(45.1)	866	(42.9)	
1	827	(27.5)	540	(26.7)	
≥ 2	826	(27.4)	614	(30.4)	
**Number of emergency visits**					0.0987
0	1353	(44.9)	860	(42.6)	
1	696	(23.1)	457	(22.6)	
≥ 2	962	(32.0)	703	(34.8)	
**Medical conditions**					
Hypertension	1449	(48.1)	866	(42.9)	0.0002
Diabetes	459	(15.2)	345	(17.1)	0.0816
Hyperlipidemia	164	(5.5)	109	(5.4)	0.9380
Mental disorders	710	(23.6)	473	(23.4)	0.8928
Ischemic heart disease	938	(31.2)	505	(25.0)	< 0.0001
Heart failure	576	(19.1)	375	(18.6)	0.6155
Liver cirrhosis	72	(2.4)	66	(3.3)	0.0622
COPD	591	(19.6)	322	(15.9)	0.0009
Renal dialysis	62	(2.1)	48	(2.4)	0.4509

*COPD* chronic obstructive pulmonary disease, *CS* cesarean section, *GA* General anaesthesia, *NA* Neuraxial anaesthesia.

Compared with AS patients who underwent neuraxial anesthesia (Table 4), those who underwent general anesthesia had higher risks of postoperative acute myocardial infarction (aOR 3.06, 95% CI 1.22–7.67), acute renal failure (aOR 1.82, 95% CI 1.11–2.98), pneumonia (aOR 1.80, 95% CI 1.32–2.46), and septicemia (aOR 1.58, 95% CI 1.18–2.12). General anesthesia was associated with an increased risk of intensive care after surgery (aOR 4.05, 95% CI 3.23–5.09). The higher medical expenditures (median 2103 vs. median 1680 US dollars, *p* < 0.0001) were also noted for patients who underwent general anesthesia compared to neuraxial anesthesia.

**Table 4:** Adverse outcomes after surgeries in patients with aortic stenosis received neuraxial and general anesthesia (N = 5031).

	NA (N = 3,011)		GA (N = 2,020)		Risk of outcomes	
	Events	%	Event	%	aOR	(95% CI)^a^
30-day in-hospital mortality	22	0.7	18	0.9	1.29	(0.67–2.48)
**Postoperative complications**						
Pulmonary embolism	8	0.3	12	0.6	1.98	(0.78–5.04)
Acute myocardial infarction	8	0.3	13	0.6	3.06	(1.22–7.67)
Acute renal failure	32	1.1	39	1.9	1.82	(1.11–2.98)
Pneumonia	89	3.0	94	4.7	1.80	(1.32–2.46)
Septicemia	95	3.2	110	5.5	1.58	(1.18–2.12)
Stroke	138	4.6	80	4.0	0.87	(0.65–1.17)
Urinary tract infection	336	11.2	216	10.7	0.86	(0.71–1.05)
Postoperative bleeding	13	0.4	15	0.7	2.03	(0.93–4.42)
Deep wound infection	11	0.4	5	0.3	0.80	(0.27–2.38)
Intensive care unitICU stay	139	4.6	285	14.1	4.05	(3.23–5.09)
Medical expenditure, USD^†^	1680 ± 1971		2103 ± 3014		p < 0.0001	
Length of hospital stay, days^†^	5.0 ± 5.0		6.0 ± 6.0		p < 0.0001	

*aOR* adjusted odds ratio, *CI* confidence interval, *GA* General anaesthesia, *NA* Neuraxial anaesthesia, *OR* odds ratio.

^a^Adjusted for all covariates listed in Table 1.

^†^Median (interquartile range).

In the stratified analyses of surgical patients with AS (Table 5), general anesthesia was associated with postoperative adverse events in females (aOR 2.28, 95% CI 1.75–2.98), males (aOR 2.84, 95% CI 2.23–3.62), and patients aged ≥ 50 years. The association between general anesthesia and postoperative adverse events was significant in patients with 0, 1, 2, and ≥ 3 medical conditions. The increased postoperative adverse events associated with general anesthesia was observed in patients with and without previous emergency visits and hospitalizations.

**Table 5:** The stratified analysis for the risk of adverse events after surgeries associated with general anesthesia (N = 5031).

		n	Adverse events*			
			Events	Rate, %	aOR	(95% CI)†
Female	NA	1313	127	9.7	1.00	(reference)
	GA	1037	185	17.8	2.28	(1.75–2.98)
Male	NA	1698	161	9.5	1.00	(reference)
	GA	983	210	21.4	2.84	(2.23–3.62)
Age 20–49 years	NA	158	13	8.2	1.00	(reference)
	GA	144	15	10.4	2.70	(0.93–7.81)
Age 50–59 years	NA	144	4	2.8	1.00	(reference)
	GA	167	15	9.0	3.66	(1.04–12.9)
Age 60–69 years	NA	436	26	6.0	1.00	(reference)
	GA	367	51	13.9	2.65	(1.55–4.55)
Age 70–79 years	NA	1013	75	7.4	1.00	(reference)
	GA	715	117	16.4	2.47	(1.78–3.43)
Age ≥ 80 years	NA	1260	170	13.5	1.00	(reference)
	GA	627	197	31.4	2.84	(2.22–3.64)
0 medical condition	NA	546	28	5.1	1.00	(reference)
	GA	466	53	11.4	3.54	(2.10–5.96)
1 medical condition	NA	966	80	8.3	1.00	(reference)
	GA	620	104	16.8	2.36	(1.70–3.28)
2 medical conditions	NA	769	80	10.4	1.00	(reference)
	GA	513	117	22.8	2.72	(1.95–3.79)
≥ 3 medical conditions	NA	730	100	13.7	1.00	(reference)
	GA	421	121	28.7	2.52	(1.83–3.45)
0 hospitalization	NA	1358	84	6.2	1.00	(reference)
	GA	866	92	10.6	2.30	(1.65–3.20)
1 hospitalization	NA	827	67	8.1	1.00	(reference)
	GA	540	96	17.8	2.94	(2.05–4.20)
≥ 2 hospitalizations	NA	826	137	16.6	1.00	(reference)
	GA	614	207	33.7	2.79	(2.13–3.64)
0 emergency visit	NA	1353	76	5.6	1.00	(reference)
	GA	860	88	10.2	2.38	(1.69–3.36)
1 emergency visit	NA	696	69	9.9	1.00	(reference)
	GA	457	88	19.3	2.38	(1.64–3.46)
≥ 2 emergency visits	NA	962	143	14.9	1.00	(reference)
	GA	703	219	31.2	2.89	(2.24–3.74)

*aOR* adjusted odds ratio, *CI* confidence interval, *OR* odds ratio, *GA* General anaesthesia, *NA* Neuraxial anaesthesia.

*Adverse events included with acute myocardial infarction, acute renal failure, pneumonia, septicemia, and intensive care unitICU stay.

^†^Adjusted for all covariates listed in Table 1.

Table S1 showed the surrogates of severity of AS on the risk of postoperative adverse events in AS patients with and without AS. The surrogates of severity of AS on the risk of postoperative adverse events in AS patients with general and neuraxial anesthesia was showed in Table S2. In Table S3, we investigated that AS patients with epidural anesthesia (aOR 1.52, 95% CI 1.05–2.20) or general anesthesia (aOR 2.73, 95% CI 2.27–3.28) had increased risk of postoperative adverse events compared with those received spinal anesthesia.

## Discussion

In this nationwide cohort study matched by propensity scores, we found that patients with AS had increased risks of 30-day mortality, pneumonia, acute renal failure, stroke, and intensive care after noncardiac surgery. Further analysis showed that AS patients who underwent general anesthesia had greater risks of pneumonia, acute renal failure, septicemia, and intensive care compared to neuraxial anesthesia. General anesthesia was significantly associated with postoperative adverse events in various subgroups. These results have important implications for anesthetic management and perioperative care in patients with AS.

In this study, we observed a modestly higher 30-day mortality rate in AS patients compared to non-AS patients after noncardiac surgery, in agreement with some reports^6,10,12,14^. Other studies showed that symptomatic AS and emergency surgery independently predicted short-term mortality in AS patients undergoing noncardiac surgery, but asymptomatic AS patients had a similar risk of perioperative mortality to controls^15,16^. These findings may be attributed to more aggressive perioperative care and hemodynamic monitoring made as a result of the increased awareness of AS hemodynamic consequences during elective surgery^15,16^. Our analyses also demonstrated that AS was independently associated with postoperative pneumonia, renal failure, stroke, and intensive care unit stay. A previous study demonstrated that only new or deteriorating heart failure was more frequent in patients with severe AS following noncardiac surgery, and the risk of other cardiovascular complications was similar between severe AS patients and controls, including myocardial infarction, stroke, and malignant ventricular arrhythmias^15^. Similarly, Agarwal et al. reported that AS-related symptoms and preexisting coronary artery disease, no AS itself, significantly predicted short-term mortality or postoperative myocardial infarction^14^. Of note, our analyses were not restricted to severe or symptomatic AS and have adjusted for the covariates of heart failure and coronary artery disease.

AS represents a distinct challenge in anesthetic management and perioperative care. Although the incidence of AS steadily increases in the aging population^2^, no randomized or prospective clinical trials looking at neuraxial anesthesia^19,20^. Previous case reports demonstrated that neuraxial anesthesia could be uneventfully performed in patients with severe AS^9,19,20^. In a retrospective analysis of 272 patients with newly diagnosed AS prior to hip fracture surgery, general anesthesia tended to be used as the severity of AS increased, but the difference in mortality rates between general and neuraxial anesthesia was not assessed^23^. Our results suggested favorable postoperative outcomes in AS patients undergoing neuraxial anesthesia, providing valuable evidence for optimal anesthesia in this population. For neuraxial anaesthesia, catheter-based epidural anaesthesia may be a safe choice for severe AS patients, allowing for a slow titration of local anesthetics and attainment of the minimum effective block level^9,18–20^. In this study, we found that patients with spinal anesthesia had the lowest risk of postoperative adverse events compared to patients with general anesthesia or epidural anaesthesia. In addition, direct arterial pressure monitoring provides beat-to-beat measurement of blood pressure and enables prompt correction of hypotension during the administration of neuraxial blockade for AS patients.

Our analysis suggested AS patients undergoing general anesthesia had higher risks of pneumonia, acute renal failure, and septicemia after surgery. First, epidemiological data showed that nearly 30% of AS patients were comorbid with chronic obstructive pulmonary disease^30,31^, a major prognostic factor for pulmonary complications after noncardiac surgery^32^. Another study reported that the use of regional anesthesia may reduce the risk of pneumonia, unplanned postoperative intubation, and prolonged mechanical ventilation in patients with chronic obstructive pulmonary disease^25^. Similarly, epidural analgesia may decrease postoperative pneumonia in patients with chronic obstructive pulmonary disease undergoing major abdominal surgery^33^. Second, a prior study reported a dose-dependent association between intraoperative tidal volumes and risk of acute kidney injury after noncardiac surgery^34^. Positive pressure ventilation increases intrathoracic pressure during inspiration, which impedes venous return and thereafter facilitates the development of acute kidney injury^35^. Third, neuraxial anesthesia may reduce the risk of surgical site infection through attenuating the stress response to surgery and inducing vasodilation and consequently enhancing tissue oxygenation^36^.

Our stratified analysis showed that the association between GA and postoperative adverse events was greater in patients with AS aged ≥ 50 but not < 50 years. We hypothesized that the older patients with AS were more likely to have multivariable coexisting diseases and were more vulnerable to surgical complications. Therefore, NA might have greater protective effect on this population.

A meta-analysis revealed the prevalence of all AS and severe AS in patients above 75 years old to be 12.4% and 3.4%, respectively^37^. Among those with severe AS, 24.4% were asymptomatic^37^. Current guidelines recommend that preoperative echocardiography be performed for patients with clinically suspected moderate or severe valvular stenosis or regurgitation^4, 5^; however, the necessity of preoperative echocardiography in patients with high cardiac risk is still a matter of debate. A recent study reported that heart failure defined by left ventricular ejection fraction < 50% was significantly associated with 90-day mortality after noncardiac surgery regardless of clinical symptoms^38^; however, another study reported that preoperative echocardiography was not linked to decreased postoperative complications or in-hospital mortality after hip fracture surgery^39^. Randomized trials are needed to assess the potential benefits of preoperative echocardiography in high-risk patients.

In this study, we investigated that the risk of postoperative adverse events associated with AS was augmented in patients with a previous aortic valve replacement. The previous observational study showed that untreated severe AS was associated with higher risks of 30-day mortality after elective intermediate-to-high-risk non-cardiac surgery, while no patient with prior aortic valve replacement died after elective non-cardiac surgery^17^. However, there was no clinical trials to demonstrate whether aortic valve intervention before elective non-cardiac surgery reduces the risk of mortality and morbidity compared to that of undertaking surgery without valve intervention.

This observational study has some limitations. First, the analysis did not include echocardiographic characteristics, such as aortic valve area, jet velocity, pressure gradient, and left ventricular ejection fraction and therefore the severity of AS cannot be determined accurately. Patients who were sicker or had more severe forms of AS might be more likely to receive general anesthesia rather than neuraxial anesthesia compared to their matched group^23^. Due to the nature of data, the information of the severity of AS was not available in the database of Taiwan’s National Health Insurance. Second, we could not differentiate between symptomatic or asymptomatic AS due to unavailability of data. Third, we have no clinical data on detailed surgical and anesthetic management, and physicians’ clinical experiences and skills that were not covered by health insurance. Fourth, contraindications to neuraxial blockade were not considered, such as bleeding tendency and severe hypovolemia. Finally, confounding by indication is possible although we have adjusted for various potential confounders. There are too many unknown variables within the neuraxial group (spinal vs epidural, intra-op management, severity of AS, use of medications, etc.) to draw any concrete conclusions.

In conclusion, AS was an independent risk factor for in-hospital complications and mortality after noncardiac surgery. Among patients with AS that received noncardiac surgeries, general anesthesia use was associated with more postoperative complications compared to neuraxial anesthesia. This study highlights the importance of anesthesia management on surgical patients with AS and suggests that neuraxial anesthesia should not be regarded as contraindicated in AS patients. However, this study may be at high risk of bias due to the retrospective nature of administrative databases and limitation in available data. Our findings warrant investigation in prospective cohorts.

## Supplementary Information

Supplementary Information.

## Abbreviations

AS: Aortic stenosis
CI: Confidence interval
ICD-9-CM: International classification of diseases, 9th revision, clinical modification
OR: Odds ratio
