# Supplementary material for: Adverse outcomes after noncardiac surgery in patients with aortic stenosis
Source: Sci Rep. 2021 Sep 30;11:19517. doi: 10.1038/s41598-021-98507-6 (PMC8484466; doi:10.1038/s41598-021-98507-6)
Supplement: Supplementary file 1 — Supplementary Information. [file 41598_2021_98507_MOESM1_ESM.doc]

| **Table S1. The surrogates of severity of AS on the risk of postoperative adverse events in AS patients with and without AS** | | | | | |
| --- | --- | --- | --- | --- | --- |
|  | Adverse events* | | | | |
|  | n | Events | Incidence, % | OR | (95% CI)^a^ |
| No aortic stenosis | 9741 | 2322 | 23.8 | 1.00 | (reference) |
| AS patients without aortic valve replacement | 9374 | 2593 | 27.7 | 1.28 | (1.19-1.38) |
| AS patients with aortic valve replacement | 367 | 131 | 35.7 | 1.50 | (1.18-1.91) |
| AS patients without hospitalization for AS | 9273 | 2550 | 27.5 | 1.27 | (1.18-1.36) |
| AS patients with hospitalization for AS | 468 | 174 | 37.2 | 1.67 | (1.35-2.07) |
| AS patients without heart failiure | 8103 | 2002 | 24.7 | 1.23 | (1.14-1.32) |
| AS patients with heart failiure | 1638 | 722 | 44.1 | 1.57 | (1.38-1.79) |
| AS patients without beta blocker | 6396 | 1675 | 26.2 | 1.26 | (1.16-1.36) |
| AS patients with beta blocker | 3345 | 1049 | 31.4 | 1.35 | (1.23-1.49) |
| AS patients without wafarin | 9614 | 2684 | 27.9 | 1.29 | (1.20-1.38) |
| AS patients with wafarin | 127 | 40 | 31.5 | 1.60 | (1.06-2.40) |
| AS patients without antiplatelet | 9241 | 2579 | 27.9 | 1.30 | (1.21-1.39) |
| AS patients with antiplatelet | 500 | 145 | 29.0 | 1.19 | (0.96-1.47) |
| AS, aortic stenosis; CI, confidence interval; OR, odds ratio.  *Adverse events include with 30-day in-hospital mortality, acute renal failure, pneumonia, stroke, postoperative bleeding and intensive care unit stay.  †Adjusted for all covariates listed in Table 1. | | | | | |

| **Table S2. The surrogates of severity of AS on the risk of postoperative adverse events in AS patients with general and neuraxial anesthesia** | | | | | |
| --- | --- | --- | --- | --- | --- |
|  | Adverse events* | | | | |
|  | n | Events | Incidence, % | aOR | (95% CI)† |
| NA without aortic valve replacement | 2906 | 278 | 9.6 | 1.00 | (reference) |
| NA with aortic valve replacement | 105 | 10 | 9.5 | 1.07 | (0.53-2.13) |
| GA without aortic valve replacement | 1920 | 374 | 19.5 | 2.62 | (2.18-3.14) |
| GA with aortic valve replacement | 100 | 21 | 21.0 | 2.42 | (1.42-4.14) |
| NA without hospitalization for AS | 2874 | 271 | 9.4 | 1.00 | (reference) |
| NA with hospitalization for AS | 137 | 17 | 12.4 | 1.28 | (0.74-2.22) |
| GA without hospitalization for AS | 1894 | 363 | 19.2 | 2.61 | (2.17-3.14) |
| GA with hospitalization for AS | 126 | 32 | 25.4 | 2.92 | (1.85-4.61) |
| NA without heart failiure | 2229 | 165 | 7.4 | 1.00 | (reference) |
| NA with heart failiure | 782 | 123 | 15.7 | 1.52 | (1.16-1.98) |
| GA without heart failiure | 1504 | 220 | 14.6 | 2.38 | (1.90-2.98) |
| GA with heart failiure | 516 | 175 | 33.9 | 4.49 | (3.46-5.83) |
| AS, aortic stenosis; CI, confidence interval; GA, general anesthesia; NA, neuraxial anesthesia; aOR, adjusted odds ratio.  *Adverse events include acute myocardial infarction, acute renal failure, pneumonia, septicemia, intensive care unit stay.  †Adjusted for all covariates listed in Table 3. | | | | | |

| **Table S3. The risk of postoperative adverse events in AS patients received general, epidural, and spinal anesthesia** | | | | | |
| --- | --- | --- | --- | --- | --- |
|  | Adverse events* | | | | |
| Patients with AS received | n | Events | Incidence, % | OR | (95% CI)† |
| Spinal anesthesia | 2684 | 245 | 9.1 | 1.00 | (reference) |
| Epidural anesthesia | 327 | 43 | 13.2 | 1.52 | (1.05-2.20) |
| General anesthesia | 2020 | 395 | 19.6 | 2.73 | (2.27-3.28) |
| AS, aortic stenosis; CI, confidence interval; OR, odds ratio.  *Adverse events included with acute myocardial infarction, acute renal failure, pneumonia, septicemia, intensive care unit stay.  †Adjusted for all covariates listed in Table 3. | | | | | |
